# Clinical effect and mechanism of aerobic exercise for knee osteoarthritis: a mini review

**DOI:** 10.3389/fphys.2025.1708750

**Published:** 2025-11-12

**Authors:** Hao-Yu Hu, Lu-Ning Jia, Wen-Yu Zhao, Sheng-Jie Guo, Zhi-Min Fang, Xi-Yue Li, Yi-Li Zheng, Pei-Jie Chen

**Affiliations:** 1 Department of Sport Rehabilitation, Shanghai University of Sport, Shanghai, China; 2 Department of Rehabilitation Medicine, Shanghai Shangti Orthopedic Hospital, Shanghai, China

**Keywords:** knee osteoarthritis, exercise therapy, aerobic exercise, biological mechanism, knee pain

## Abstract

Knee osteoarthritis (KOA) is a prevalent degenerative joint disease characterized by pain, dysfunction, and stiffness, significantly impairing quality of life. While various interventions exist, aerobic exercise stands out as a safe and effective core treatment. This review synthesizes current evidence on the therapeutic benefits and underlying mechanisms of aerobic exercise for KOA. We recommend low-to-moderate intensity aerobic training (RPE 11–14) for KOA patients, performed 3–4 times per week for 30–60 min, for at least 6 weeks. Recommended modalities include gentle exercises like Wuqinxi, Baduanjin, and yoga, or water-based exercises and swimming, which can offer additional benefits for weight management. The therapeutic effects of aerobic exercise on KOA are multifaceted. Mechanistically, it modulates inflammatory responses by balancing pro- and anti-osteoclastogenic cytokines and inhibiting inflammatory signaling pathways, thereby alleviating pain and promoting cartilage repair. Additionally, aerobic exercise contributes to weight control, reducing knee joint load and improving cartilage health. It also provides appropriate mechanical loading to facilitate osteogenesis and preserves muscle mass, particularly in the lower extremities, mitigating muscle loss and reducing joint pressure. Despite these benefits, the precise exercise modalities, patterns, and intensities for different KOA grades remain to be fully defined and require further clinical validation. Future research should focus on quantifying exercise prescriptions to optimize anti-inflammatory effects, muscle preservation, and cartilage regeneration, as well as exploring the potential of combining aerobic exercise with other training types to enhance outcomes.

## 1 Introduction

Knee osteoarthritis (KOA) is a prevalent degenerative joint disease characterized by cartilage regression, osteophyte formation, and subchondral bone sclerosis (Primorac et al., 2020). The prevalence rate of KOA in the elderly population is more than 50%. Women have higher prevalence than men, with knee dysfunction rates reaching 53% (Zhang et al., 2020). The major clinical symptoms of KOA are knee joint pain, dysfunction, and stiffness, which can significantly impair knee function and reduce quality of life. Risk factors for KOA include gender, age, obesity, lack of exercise, and previous knee injury (Martel-Pelletier et al., 2016). These factors can exacerbate the patient’s clinical symptoms. Previous studies demonstrated that KOA imposes a substantial economic burden in terms of medical costs (Mesa-Castrillon et al., 2021). Furthermore, all patients diagnosed with KOA can anticipate an effective and cost-efficient solution for the treatment of their pain and dysfunction.

The clinical interventions for KOA include pharmacotherapy, total knee arthroplasty, knee bracing, and exercise therapy (Surakanti et al., 2023). However, Pharmacological treatment of KOA offers pain relief and improved function, but its long-term use may be limited by side effects and does not address the underlying disease progression (Kan et al., 2019). The use of knee braces is effective in reducing short-term pain for patients with KOA. However, prolonged use may result in quadriceps femoris weakness and muscle atrophy. Among various interventions, exercise therapy has been proven to be one of the core acceptable and safe clinical approach for reducing knee pain intensity and dysfunction (Goh et al., 2019; Gay et al., 2016; Deng et al., 2023; Ferreira et al., 2015).

Exercise therapy primarily comprises aerobic exercise (AE), hydrotherapy, joint mobilization, muscle stretching, and resistance training (Zeng et al., 2021). However, high-intensity resistance training combined with hydrotherapy may exacerbate knee pain and increase the risk of adverse events (Waller et al., 2017). Additionally, patients with moderate to severe KOA often experience discomfort around the patella during closed kinetic chain exercises that involve a large range of motion in the knee joint, such as deep squats (Lin et al., 2022). However, most studies have indicated that patients with KOA experience decreased knee pain and improved knee function when they engage in long-term aerobic exercises, such as cycling, swimming, low-intensity running, and jogging (Alkatan et al., 2016; Kabiri et al., 2018). Aerobic exercise is a non-invasive stimulation widely used to treat patients with KOA because of its many benefits, including lack of side effects, suitability for the elderly population, and convenience. The purpose of this study is to investigate the therapeutic benefits and underlying mechanisms of aerobic exercise in treating patients with KOA.

## 2 Specific exercise prescription for patients with KOA in aerobic training

Aerobic exercise is effective in improving joint pain, stiffness, and swelling in patients with KOA, thereby improving their aerobic function, physical function, level of function, and quality of life and alleviating their emotional and psychological wellbeing. In this review, the keywords “aerobic exercise” and “knee osteoarthritis” were used to search the literature of the last decade in the pubmed database and web of science database. Table 1 shows the clinical effects of aerobic exercise on KOA.

**TABLE 1:** The clinical effects of aerobic exercise on KOA.

Study		Participants				Intervention						Result	
						Intervenion group			Control group				
Author, year	Design	Population	Sample size	Age	Gender (F/M)	Type	Frequency and Duration	Intensity	Type	Frequency and Duration	Intensity	Outcomes measure	Clinical effects
Lin et al. (2022)	RCT	Older adults with mild KOA	38 (84% completed) and 7 dropped outs (16%)	CRE:75.6 ± 4.4 years COM:76.0 ± 5.6 years	CRE F:19 M:1 COM F:16 M:2	Comfortably on a slide seat with both feet on the foot platforms of the computer-aided rowing exercise system	That rowing exercise system 30-min in each Section 2/week for 12 weeks	The target force for triggering the game was set at 50% of 1-RM during the first week and then progressively increased to 5% of the original 1-RM every 2 weeks	Received regular resistance exercise programs	2/week for 12 weeks	—	WOMAC muscle strength 10-m walk tests	Participants’ functional fitness in the CRE group exhibited significantly higher adjusted mean post-tests scores than those in the CON group after the intervention
Xiao et al. (2021)	RCT	Community dwelling older adults with KOA	68 (91.84% completed) 0 dropped out (0)	70.95 ± 9.85 years	F:43 M:25	The WQX exercise programme for KOA consists of three parts: warming up, WQX exercise and cooldown	WQX exercise 4/week 60min/session	—	Gradual increase in training intensity, knee load and exercise difficulty during the program	4 days weekly for 12 weeks; The aerobic training: lasted 30 min	Aerobic training: between 6 and 12 RM (repetition maximum) performed at 75%–85% of heart rate	BBS 6MWT TUG 30s STS WOMAC	The WQX group maintained or improved in all nine measures from post-test to follow-up, whereas the control groupmsignificantly declined in WOMAC pain, Knee extensor strength and Knee flexor strength
Waller et al. (2017)	RCT	Post-menopausal women with mild knee osteoarthritis	87 (87.36%completed) and 11 dropped outs (12.64%)	60–68years	F:87	An aquatic resistance training sessions	1 h, 3 times a week for 16 weeks (48 sessions in total)	Intensity with three resistance levels; barefoot, small resistance fins and large resistance boots	Maintainusual care and were asked to continue their usual leisure time activities	1 h,during the 4-month intervention period	Light stretching, relaxation and social interaction	VAS DXA KOOS self-reported diaries	High intensity aquatic resistance training decreases fat mass and improves walking speed.Only improvements in walking speed were maintained at 12-month follow-up. Higher levels of LTPA were associated with fat mass loss
Torstensenet al. (2023)	RCT	Patients with diagnosed knee osteoarthritis and a history of pain and decreased knee function	189 (77%completed) and44 dropped outs (23%)	45–85years	F:106 M:83	high-dose therapy 2.low-dose therapy aerobic ergometer cycling local (joint-specific) exercises	11 exercises; 70–90 min; 5 exercises; 20–30 min; All participants exercised 3 times per week for 12 weeks, for a total of 36 treatments	Global (aerobic) Semiglobal (multisegmental) Local (joint-specific) exercises	—	—	—	KOOS VAS EQ-5D	Both groups improved over time, but there were no benefits of high-dose therapy in most comparisons
Schlenket al. (2021)	RCT	Adults with knee osteoarthritis and hypertension	182 (94.51%completed) and10 dropped outs (5.49%)	Older than 50 years	F:133 M:49	The STAR group:1.LEE, and fitness walking; 2.telephone sessions 3.e-diary for self-monitoring physical activity	6 weekly 60–65 min; nine biweekly 15–20 min; daily. During the 6-month	—	The attention-control group:received usual care and telephone sessions	Six weekly and nine biweekly 15–20 min.For 6 months	—	e-diary GT3X ActiGraph accelerometer automatic professional digital BP monitor SPPB WOMAC	Physical activity interventions can promote physical activity and improve outcomes in adults with KOA.The STAR group had better knee function at immediate postintervention and 6-month postintervention compared with baseline
Kabiri et al. (2018)	RCT	Grade 2 or 3 of knee OA	78 (89.74%completed) and 8 dropped outs	Older than 40 years	F: 56 M: 14	Aerobic exercise (Treadmill, Cycle ergometer, Arm ergometer) with Resistance training and Stretching exercise	Aerobic exercise and Resistance training: 3/week 30 min/session Stretching exercise 3-5stretches/ muscle group; hold 20–30 s	Aerobic exercise: Borg RPE scale: 11–13 Resistance training: 40%–60% 1RM Stretching exercise: Stretch to full range of motion	No intervention	—	—	VAS 6MWT Timed up and go test 30s STS KOOS	Significant improvement in pain and functional status
Marconcin et al. (2017)	RCT	Knee osteoarthritis	80 (83.75%completed) and 67 dropped outs (16.25%)	Age ≥60 years	The SMEG F: 19 M: 13 The, E.G., F: 28 M: 7	The SMEG (the treatment group) included self-management and exercise with glucosamine and chondroitin sulfate supplement	90-min intervention (30 min self-management and 60 min exercise)twice a week for 12 weeks	—	The, E.G., (the control group) received a book 12 telephone calls three education sessions with glucosamine and chondroitin sulfate supplement	Twice a week for 12 weeks	—	KOOS EQ-5D-5F VAS 6MWT CSR BST Handgrip test FRSTST	The PLE2NO self-management and exercise interven-tion had a significant group effect in favor of the intervention group in self-management behavior (CWP) and health-related physical fitness out-comes (functional lower limb strength and aerobic capacity)
Samut et al. (2015)	RCT	Patients with grade 2–3 knee OA	42 (isokinetic exercise group n = 25; aerobic group n = 14; control group n = 13)	Older than 50 years	Ninety percent of the patients were female	1.aerobic group (aerobic exercise on a treadmill) 2.isokinetic exercise group (in biodex isokinetic system)	3 days/week for 6 weeks	1.aerobic group:65 %-70% of age related heart rate for the first 4 weeks and 70%–75% for the next 2 weeks 2.isokinetic exercise group: performed 5 concentric flexion and extension at angular velocities of 60^0^/s, 90^0^/s, 120^0^/s and 180^0^/s	After baseline evaluation control group was informed about the disease and recommendations about precautions to be taken	—	—	VAS WOMAC 6MWT 30 s STS	Significant decrease in VAS and WOMAC scores, and significant increase in functional capacity and muscle strength in both exercise groups compared with the control group
Cheunget al. (2016)	RCT	Patients with OA	83(Retention rate was 82%)	Age 71.6 ± 8.0 years	84% female	1.HY group: poses in the seated, supine, prone, and standing positions; breathing exercises, and relaxation/mindfulness training and key yoga poses 2.ASE group:mild aerobic exercise、isometric (without moving the joints) and isotonic (moving the joints) exercises	Both HY and ASE groups involved 8 weekly 45-min group classes with 2–4 days/week home practice sessions	1.HY group:each class consisted of approximately 8–10 yoga poses with a 2–3 new, variable poses were introduced at each session 2.from mild to strengthening exercises	Control group received OA education brochures and phone calls from study staff	weekly	—	WOMAC 5-point Likert SPPB	Both HY and ASE improved symptoms and function but HY may have superior benefits for older adults with knee OA.
de Rooij et al. (2017)	RCT	Patients with knee osteoarthritis (KOA) and comorbidities	126 (85.71%completed) and 9 dropped outs (14.29%)	Intervention group:63.2 + 8.4 control group:63.9 + 12.4	F:95 M:31	muscle-strength training of the lower limb and aerobic training	Two sessions of 30–60 min a week during 20-week	Low training intensity (Borg scale ≤11) moderate training intensity (Borg scale 12–14) high training intensity (Borg scale ≥15)	Received their current medical care for KOA and comorbid disease	32 weeks	—	WOMAC 6MWT	At 30months follow, the mean improvements in the intervention group were 33% on the WOMAC scale and 15% on the 6MWT.
Alkatanet al. (2016)	RCT	Sedentary middle-aged and older adults with OA	48 (83.33%completed) and 8 dropped outs (16.67%)	Swimming group:61 ± 1 cycling group:59 ± 2	F:44 M:4	Swimming、cycling	45 min/day, 3 days/week at 60%–70% heart rate reserve for 12 weeks	Initially,at an exercise intensity of 40%–50% of heart rate reserve (HRR); ultimately,t an intensity of 60%–70% of HRR	—	—	—	WOMAC 5-point Likert maximal handgrip strength isokinetic knee extension flexion power 6-min walk test	Regular swimming exercise reduced joint pain and stiffness associated with OA and improved muscle strength and functional capacity in middle-aged and older adults with OA.
An et al. (2013)	Trial	Patients with KOA	28 (78.57%completed) and 6 dropped outs (21.43%)	55–82 years	The patients who completed the trial F:19 M:3	The Baduanjin exercise	Sessions were held for 30 min five times a week for 1 year	—	—	—	—	WOMAC SF-36 6-MWT ISKEF BMI	The oneyear exercise of Baduanjin is a safe and feasible option for knee OA in Chinese. It offers reductions in pain, stiffness, disability, and improvement in general and emotion health. It also reduces BMI, enhances the knee extensors and flexors strength, and improves the patients' aerobic ability
Koli et al. (2015)	RCT	Postmenopause women with KOA	72 (90%completed) and 8 dropped outs	50–65 years	All female	High-impact multi-directional modified aerobic and step-aerobic jumping exercise programs alternated every 2 weeks Supervised group exercise classes	lasting 55 min, were carried out three times a week for 12 months. Loading was gradually increased after 3 months by progressively raising	—	—	—	—	T2 relaxation time was determined using a Siemens Magneton Symphony Quantum 1.5-T scanner Maximal isometric knee extension was measured in sitting position with a knee angle of 60°, using a dynamometer chai Leg extensor power in each leg was measured with the Nottingham Power Rig Cardio respiratory fitness	Our 12-month randomized controlled high-impact exercise trial in postmenopausal women with mild OA showed decreased mean T2 relaxation time, indicating improved patellar cartilage quality. In addition, physical performance improved

Abbreviations: F, famale; M, male; RCT, randomized controlled clinical trial; KOA, knee osteoarthritis; CRE, computer-aided rowing exercise; COM, control group; RM, repetition maximum; WOMAC, the Western Ontario and MacMaster Universities; WQX, wuqinxi; BBS, berg balance scale; 6MWT, 6 min walking test; TUG, timed up and go test; VAS, visual analogue scale; DXA, the dual-energy X-ray absorptiometry; KOOS, the knee injury and osteoarthritis outcome score; LTPA, leisure time physical activity; EQ-5D, the EuroQol Group 5-Dimension; STAR, staying active with arthritis; LEE, lower-extremity exercise; SPPB, short physical performance battery; SEMG, the Self-Management and Exercise Group; E.G., the Educational Group; EQ-5D-5F-VAS, EuroQol five-dimension five-level visual analog scale; CSR, chair sit-and-reach; BST, back scratch test; FRSTST, five repetition sit-to-stand test; CWP, the communication with physician; 30s STS, 30 s sit to stand test; HY, hatha yoga; ASE, aerobic/strengthening exercises; SF-36, Study Short Form-36; ISKEF, the Isokinetic Strength of the Knee Extensors and Flexors; BMI, body mass index.

### 2.1 Timing and frequency of aerobic exercise

In the reviewed literature, the duration of exercise interventions ranged from 6 to 20 weeks. Rooij et al. studied 126 patients diagnosed with KOA, who underwent aerobic training sessions lasting for 30–60 min twice a week for 20 weeks (de Rooij et al., 2017). Gulbuz Samut et al. conducted aerobic treadmill training three times a week for 6 weeks on patients with grades 2–3 KOA (Samut et al., 2015). The weekly training frequency ranged from 2 to 6 times in 12 studies that met the requirements. Among them, seven studies prescribed a weekly training frequency of three times (Waller et al., 2017; Torstensen et al., 2023; Kabiri et al., 2018; Samut et al., 2015; Alkatan et al., 2016; An et al., 2013; Koli et al., 2015), while one paper did not specify the frequency (de Rooij et al., 2017). Schlenk et al. conducted a training program for 182 patients with KOA, focusing on lower limb walking exercises performed six times a week (Schlenk et al., 2021). However, all 12 studies included in the analysis maintained a controlled duration of aerobic exercise between 30 and 90 min. Torstensen et al. categorized the training duration into high and low doses, with each session lasting 70–90 min for high-intensity dose and 20–30 min for low-intensity dose (Torstensen et al., 2023). The time of a single training session was not specified by Samut et al. in their description (Samut et al., 2015). In summary, we recommend a training frequency of 3–4 times per week, with each session lasting 30–60 min for patients with KOA undergoing aerobic training, and the program should be continued for at least 6 weeks.

### 2.2 Methods of aerobic exercise

Aerobic training has various modalities. Among the 13 studies that investigated aerobic exercise interventions in KOA, three studies utilized power cycling and treadmill training protocols (Kabiri et al., 2018; Samut et al., 2015; Alkatan et al., 2016). Two studies incorporated traditional Chinese training methods, including Baduanjin and Wuqinxi (Xiao et al., 2021; An et al., 2013). Additionally, two studies employed water-based exercises and swimming as intervention strategies (Alkatan et al., 2016; Waller et al., 2017). In three cases, a combination of aerobic exercise and strength training was implemented (de Rooij et al., 2017; Cheung et al., 2017; Kabiri et al., 2018). One study utilized computer-assisted rowing training, while another focused on lower limb walking exercises (Schlenk et al., 2021).

The pain and knee joint function of patients with knee arthritis was alleviated by low-intensity aerobic training and high-intensity training (Torstensen et al., 2023). Exercises such as Baduanjin, Wuqinxi and yoga are considered safe, slow, low-intensity aerobic exercises (Xiao et al., 2021; An et al., 2013; Cheung et al., 2017). Researchers conducted exploratory studies on patients' mental health, depression anxiety, and mood and obtained positive results compared with other aerobic exercises. This finding seems to be related to the fact that they are safe, slow multicomponent exercises. These types of exercises are gentle and can be adapted to the needs and limitations of older adults. They may be safer exercise options for those with functional limitations because of OA or other pre-existing musculoskeletal conditions (Chan et al., 2017; Cui et al., 2019).

In addition, the current results suggest that non-weight-bearing exercise in water may reduce joint pain and stiffness experienced by patients with OA during daily activities on land (Waller et al., 2017). Adherence to exercise is a common issue in terms of aerobic interventions for people with KOA; in the literature reviewed, older adults are adherent to exercises that are simple, moderately physically demanding, and not boring (Lin et al., 2022). For example, aquatic exercises have higher adherence than land-based exercise, and the high adherence of the WQX group may be attributed to the fact that WQX exercise is of low to moderate intensity and is very safe because physical exertion required does not even burden older adults (Tao et al., 2024). Its elegant postures and movements are relatively simple to memorize and can motivate learning and practice (Tao et al., 2024; Guo et al., 2022).

In conclusion, we recommend the adoption of low-intensity training methods such as Wuqinxi, Baduanjin, and yoga for elderly patients with KOA. Furthermore, water-based exercises or swimming may offer greater benefits compared with land-based training in terms of weight management for patients with KOA, thereby reducing knee pain and enhancing functionality.

### 2.3 Intensity of aerobic exercise

Seven of the 12 studies examined explicitly provided specific measures of intervention intensity. Among them, two studies utilized heart rate-related indicators such as age-related maximum aerobic heart rate and heart rate reserve (HRR) (Samut et al., 2015; Alkatan et al., 2016). Additionally, three studies employed self-perceived fatigue scales, such as RPE index or Borg rating of perceived exertion to evaluate training intensity (de Rooij et al., 2017; Kan et al., 2019; Segal et al., 2012). Rooij et al., classified training intensity into three distinct tiers: low, moderate, and high, with Borg scores of below 11, 12–14, and higher than 15, respectively. The remaining studies employed a proportion of 1-RM as a determinant of training intensity. Most exercise programs used moderate-intensity aerobic training (RPE maintained at 11–14, 60%–75% of maximal heart rate), often with progressive aerobic training, namely, a gradual transition from low-intensity to moderate–high-intensity (de Rooij et al., 2017). Some studies compared low and high doses and reported improvement over time in both groups; in most comparisons, high-dose treatment had no benefit (Torstensen et al., 2023). However, a high-intensity water training showed progressively implemented high-impact and intensive exercise and provided adequate stimuli; as such, it had favorable effects on patellar cartilage quality and general health/physical function in patients with mild knee OA (Waller et al., 2017).

In conclusion, we recommend patients with senile osteoarthritis to engage in low-to moderate-intensity aerobic training. The specific training intensity can be determined using the Rate of Perceived Exertion (RPE) scale, ranging from 11 to 14 for aerobic exercises. Adopting this prescribed intensity of training can effectively alleviate KOA pain and improve knee joint function.

## 3 Biological mechanism of aerobic exercise for osteoarthritis

A plethora of research indicates that engaging in aerobic exercise enhances cardiorespiratory function and peripheral circulation, stimulates body fat metabolism, optimizes muscle utilization, and mitigates muscular atrophy (Ferreira et al., 2015; Gay et al., 2016). Moderate aerobic exercise can augment synovial fluid production, thereby ameliorating pain caused by joint friction (van der Kraan et al., 2017; Musumeci et al., 2015). During aerobic exercise, the lower extremity muscle groups can be strengthened, which in turn reduces the load on the knee joint and enhances knee mobility function (Shorter et al., 2019). This phenomenon ultimately leads to an improved quality of life for patients with KOA. For KOA patients without aerobic exercise, on the one hand, it will reduce the metabolism of adipose tissue, making the patients further overweight, which in turn will increase the load on the joints, joint pain and dysfunction; on the other hand, the reduction of joint movement will also lead to muscle weakness, muscle atrophy, and stiffness of the joints. Both of the above problems will make the patient even more unable to perform aerobic exercise, thus creating a vicious circle (Supplementary Figure 1).

### 3.1 Inflammation response and associated inflammatory mediators

**FIGURE 1:**
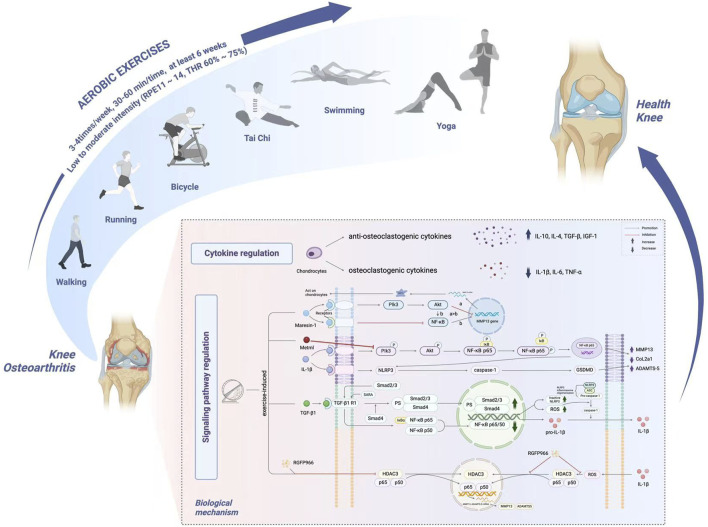
The optimal index and mechanism of aerobic exercise intervention in knee osteoarthritis. By combining all the reviewed literature, we recommend that patients train for 30–60 min for three to four sessions per week for at least 6 weeks. The recommended RPE of aerobic exercise for training intensity ranges from 11 to 14, Target heart rate maintained at 60%–75%. The biological mechanisms underlying the therapeutic effects of aerobic exercise on knee osteoarthritis are multifaceted. At the cellular level, aerobic exercise promotes the upregulation of anti-osteoclastic cytokines, including IL-10, IL-4, TGF-β, and IGF-1, while downregulating pro-osteoclastic cytokines such as IL-1β, IL-6, and TNF-α. At the signaling pathway level, exercise mediates anti-inflammatory effects through various mechanisms: (1) Maresin-1 exerts therapeutic effects on fibroblast-like synoviocytes by activating the PI3K/AKT pathway to inhibit MMP-13 secretion and suppressing the NF-κB pathway; (2) Exercise-induced metrnl reduces inflammation and inflammation-related pyroptosis in IL-1β-treated chondrocytes by inhibiting the PI3K/Akt/NF-κB and NLRP3/caspase-1/GSDMD pathways; (3) Mechanical stress increases TGF-β1, which binds to TGF-β1 type I receptors to activate Smad2/3 and inhibit NF-κB signaling, thereby reducing pyroptosis through coordinated nuclear translocation of Smad2/3 and suppression of NF-κB p65; and (4) Moderate-intensity exercise inhibits the nuclear translocation of the HDAC3/NF-κB complex, decreasing inflammatory protein expression, while the HDAC3 inhibitor RGFP966 further alleviates osteoarthritis-associated inflammation via this pathway (This figure was created with BioRender.com).

Aerobic exercise has therapeutic effects on KOA, including reducing pain, improving functional activity, protecting chondrocytes and promoting cartilage repair by regulating cytokine profile and inhibiting pro-inflammatory signaling pathways, while activating anti-inflammatory signaling pathways and mechanosensitive signaling pathways. The specific adjustment mechanism will be shown in Figure 1. Furthermore, exercise activates mechanosensitive signaling pathways, as evidenced by Cavallero et al., who demonstrated that aerobic exercise activates a mechanosensitive transcriptome leading to the production of anti-inflammatory metabolites (Cavallero et al., 2023). They demonstrated that exercise activates a mechanosensitive transcriptome, uncovering endothelial SCD1-catalyzed anti-inflammatory metabolites. This suggests that exercise-induced mechanical stimuli can trigger protective responses at the molecular level.

#### 3.1.1 Regulation of cytokine profiles and pain perception

The role of osteoblasts and osteoclasts in the pathogenesis of KOA is complex and multifaceted, with exercise playing a pivotal role in modulating their activities through the production of cytokines. Exercise not only influences bone metabolism but also impacts pain perception in patients with KOA. By regulating the balance between osteoclastogenic and anti-osteoclastogenic cytokines, exercise can significantly alter the bone microenvironment, thereby affecting the progression of the disease.

Aerobic exercise has been shown to regulate the production of osteoclastogenic cytokines, which are known to stimulate the differentiation and activation of osteoclasts, the cells responsible for bone resorption. Cytokines such as interleukin-1β (IL-1β), interleukin-6 (IL-6), and tumor necrosis factor alpha (TNF-α) are well-documented osteoclastogenic cytokines. Smith et al. reported that long-term exercise can modulate the production of these cytokines by peripheral blood mononuclear cells (Smith et al., 2016). Specifically, aerobic exercises like running and cycling have been found to reduce serum levels of IL-6 and TNF-α in patients with KOA (Samut et al., 2015). This reduction in inflammatory cytokines is believed to be one of the mechanisms behind the pain-relieving effects of exercise in KOA patients (Susko and Fitzgerald, 2013). Furthermore, IL-1β, another osteoclastogenic cytokine, has been shown to be modulated by exercise in articular cartilage, favoring an anti-inflammatory cytokine profile that may help to suppress osteoclast activity and bone resorption (Rojas-Ortega et al., 2015).

On the other hand, anti-osteoclastogenic cytokines play a crucial role in inhibiting osteoclast differentiation and activation, thereby promoting bone formation and maintenance. Cytokines such as interleukin-10 (IL-10) and interleukin-4 (IL-4) are known to possess anti-osteoclastogenic properties. IL-10, in particular, has been shown to inhibit osteoclastogenesis by suppressing the expression of RANKL, a key factor in osteoclast differentiation (Smith et al., 2016). Exercise has been reported to increase the production of IL-10 in articular cartilage, which may contribute to its protective effects on bone metabolism in KOA patients (Rojas-Ortega et al., 2015). Additionally, other cytokines such as transforming growth factor-β (TGF-β) and insulin-like growth factor-1 (IGF-1) have also been shown to possess anti-osteoclastogenic properties and their levels can be modulated by aerobic exercise, further influencing bone metabolism in KOA.

In conclusion, the balance between osteoclastogenic and anti-osteoclastogenic cytokines is crucial in maintaining bone health and preventing the progression of KOA. Aerobic exercise plays a significant role in modulating the production of these cytokines, thereby influencing bone metabolism and pain perception in patients with KOA. By promoting an anti-inflammatory cytokine profile and inhibiting osteoclast activity, exercise may help to slow down the progression of the disease and improve the quality of life of patients with KOA. Future studies are needed to further elucidate the mechanisms underlying the effects of exercise on cytokine production and bone metabolism in KOA, and to develop more effective exercise interventions for the management of this chronic condition.

#### 3.1.2 Modulation of inflammatory signaling pathways

Aerobic exercise also demonstrates its therapeutic efficacy through the modulation of the inflammatory signaling cascade. Liu et al. demonstrated that treadmill exercise-induced meteorin-like protein protects chondrocytes from inflammation and pyroptosis by inhibiting the PI3K/Akt/NF-κB and NLRP3/caspase-1/GSDMD signaling cascades (Liu et al., 2023). Similarly, Lu et al. showed that maresin-1, upregulated by treadmill exercise, suppresses IL-1β-induced MMP-13 secretion in OA synovioblasts by activating PI3K/AKT and inhibiting NF-κB pathways (Lu et al., 2021). Wang et al. further corroborated this by highlighting the protective role of mechanical stress against chondrocyte pyroptosis via TGF-β1-mediated Smad2/3 activation and NF-κB inhibition (Wang et al., 2023). These findings underscore the pivotal role of aerobic exercise in dampening inflammatory responses in KOA.

Cartilage homeostasis also plays a crucial role in KOA, and aerobic exercise modulates the expression of genes and signaling molecules critical for cartilage homeostasis. Zhang et al. revealed that treadmill exercise inhibits the HDAC3/NF-κB pathway, thereby exerting therapeutic effects on OA in rats (Zhang et al., 2019). Liu et al. observed abnormal expression of key genes in the Wnt/β-catenin pathway in an exercise-induced OA rat model, suggesting exercise-mediated alterations in chondrogenic differentiation and cartilage repair (Liu et al., 2016). Rojas-Ortega et al. found that exercise modulates the expression of IL-1β and IL-10 in articular cartilage, favoring an anti-inflammatory environment (Rojas-Ortega et al., 2015). These studies indicate that aerobic exercise modulates gene expression profiles, influencing cartilage integrity and OA progression.

### 3.2 Weight control and cartilage improvement

Obesity is a significant risk factor for osteoarthritis, and its pathogenesis involves intricate endocrine mechanisms (Collins et al., 2021). Weight loss is associated with decreased medial cartilage volume and improved knee symptoms, whereas weight gain is associated with decreased medial cartilage volume and worsened knee symptoms (Stürmer et al., 2000). These results suggest that in obese individuals, small changes in body weight may have disease-modifying effects on knee joint structure and symptoms (Misra et al., 2019). Although weight loss is an important primary treatment strategy for patients with obesity, avoiding further weight gain should also be a clinical goal.

Weight management can be effectively achieved with the incorporation of aerobic exercises. Swimming, running, Wuqinxi (a kind of Chinese traditional aerobic exercise), and other forms of physical activity can reduce body weight among intervention groups (Xiao et al., 2021; Alkatan et al., 2016; Schlenk et al., 2021). Furthermore, aerobic training is an optimal method for alleviating knee joint pressure pain.

### 3.3 Mechanical loading facilitates osteogenesis

Aerobic exercise regimens for individuals with KOA typically involve activities, such as jogging, swimming, aquatic sports, Tai Chi, cycling, and Baduanjin (antoher kind of Chinese traditional aerobic exercise) (An et al., 2013; Alkatan et al., 2016; Lin et al., 2022; Kabiri et al., 2018). These exercises are predominantly gentle and rhythmically repetitive in nature and provide targeted pressure stimulation to the surface of the knee cartilage in patients with KOA. Moreover, moderate-intensity aerobic exercise is an effective form of aerobic training for improving pain and function in patients with KOA (Zeng et al., 2021). Appropriate osseous stress can stimulate osteoblast secretion (Smith et al., 2016). A study discovered that applying a specific level of bone pressure to patients could promote cartilage repair and reduce knee joint pain. Iijima reported that appropriate osteostimulation in mice with KOA can alleviate knee pain and enhance their functionality through the application of specific levels of osteogenic pressure compared with those who remain in a prolonged static state (Iijima et al., 2017).

### 3.4 Improved muscle preservation

An epidemiological investigation of patients with KOA revealed that nearly half of them experienced a decline in quadriceps muscle strength and had low limb muscle circumference (Baumgartner et al., 1998). Studies indicated a negative correlation between quadriceps muscle strength and knee pain rating in patients with KOA (Latham and Liu, 2010; Segal et al., 2012). Meanwhile, this muscle weakness is not only a consequence of KOA but also a contributing factor, as it exacerbates joint stress and pain. In the context of aging, this decline in muscle strength is particularly concerning, as the prevalence of sarcopenia, which includes a reduction in muscle cross-sectional area and decreased muscle strength, affects approximately 30% of individuals aged 60 years and above (Petermann-Rocha et al., 2022). Aerobic training can facilitate isotonic resistance contraction of the major lower limb muscle groups through joint movement, thereby mitigating muscle content loss (Ferreira et al., 2015; Gay et al., 2016). Furthermore, aerobic training may reduce sarcopenia incidence among older adults. Regular aerobic exercises, such as running and swimming, can enhance joint lubrication and increase muscle mass in older adults (Alkatan et al., 2016). In summary, aerobic training can preserve the muscular strength of elderly individuals, particularly the major muscles of the lower extremities. This phenomenon mitigates muscle loss and reduces pressure on knee joints caused by weakened musculature, ultimately alleviating knee pain.

## 4 Future prospects

Based on current evidence, the following directions for further research on exercise interventions for KOA populations are recommended: (1) aerobic exercise intensity and frequency ranges that promote anti-inflammatory factor production as well as reduce inflammatory factor production; (2) aerobic training that promotes isotonic resistance contraction of the major muscle groups of the lower extremity and reduces the rate of loss of muscle mass; (3) studies on the mechanisms by which aerobic exercise significantly increases the growth of damaged cartilage in patients with rapid KOA; (4) a quantitative exercise program for patients with different grades of KOA; and (5) the possibility of combining aerobic exercises with other types of training to improve the outcome of KOA.

## 5 Conclusion

Evidence indicates that low-to moderate-intensity aerobic exercise modulates osteoclastogenic and anti-osteoclastogenic cytokines, influences inflammatory signaling pathways, and thereby alleviates joint pain, stiffness, and swelling, while enhancing aerobic capacity, physical performance, and quality of life in patients with KOA. Based on the reviewed literature, we recommend training for 30–60 min, three to four sessions per week, for at least 6 weeks, with an RPE of 11–14. The potential biological mechanisms of aerobic intervention in KOA include: (1) regulation of inflammatory responses and mediators; (2) weight control and cartilage preservation; (3) mechanical loading promoting osteogenesis; and (4) improved muscle maintenance. Nevertheless, the optimal exercise modalities, patterns, and intensities remain undefined and require further clinical validation. Quantitative exercise prescriptions tailored to different KOA grades are also lacking, highlighting the urgency of future research.
