# Supplementary material for: Clinical effect and mechanism of aerobic exercise for knee osteoarthritis: a mini review
Source: Front Physiol. 2025 Nov 12;16:1708750. doi: 10.3389/fphys.2025.1708750 (PMC12646927; doi:10.3389/fphys.2025.1708750)
Supplement: Supplementary file 1 [file Supplementaryfile1.docx]

Supplementary Material

# Supplementary Figures

**
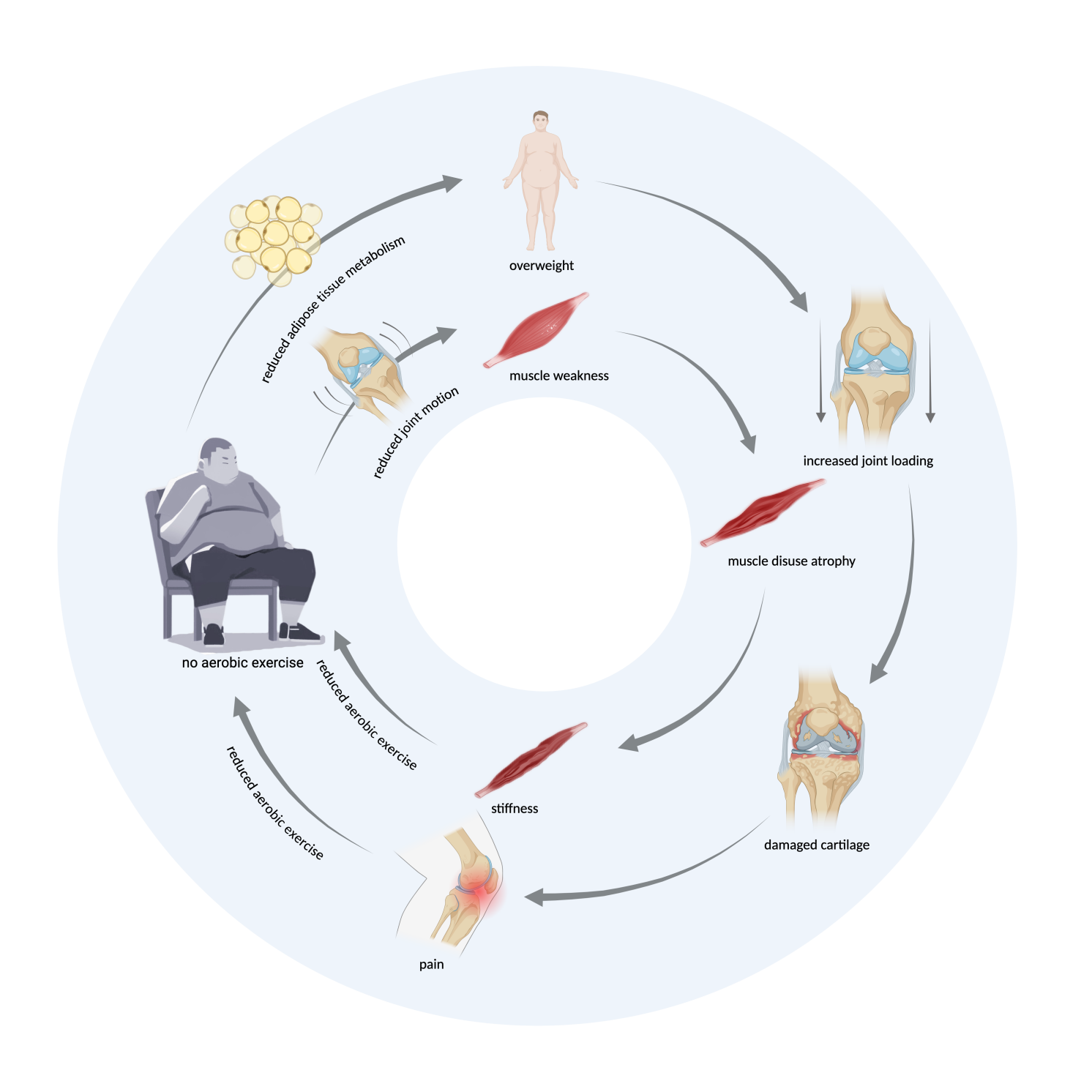
**

**Supplementary Figure 1.** The vicious circle caused by no aerobic exercise. (This figure was created with BioRender.com)

# Supplementary Tables

| Exercise Modality | Author, year | Sample size | Key Findings (Direction of Effect) | |
| --- | --- | --- | --- | --- |
|  |  |  | Functional improved | Pain decreased |
| Land-Based Aerobic | Pei-Ling Lin et al（2022） | 38(84% completed) | 1 | 0 |
|  | Chun Mei Xiao et al （2021） | 68（91.84% completed） | 1 | 1 |
|  | Elizabeth A. Schlenk et al (2021) | 182 (94.51%completed) | 1 | 0 |
|  | Sanaz Kabiri et al (2018) | 78 (89.74%completed) | 1 | 0 |
|  | Rooij et a l(2017) | 126 (85.71%completed) | 1 | 0 |
|  | Mohammed Alkatan (2016) | 48 (83.33%completed) | 1 | 1 |
|  | Corjena Cheung et al（2016） | 83 (Retention rate was 82%) | 1 | 0 |
|  | AN Bing-chen (2013) | 28 (78.57%completed) | 1 | 1 |
| Aquatic Therapy | Waller et al (2017) | 87 (87.36%completed) | 1 | 1 |
|  | Mohammed Alkatan (2016) | 48 (83.33%completed) | 1 | 1 |
| High-Intensity Training | Tom Arild Torstensen et al (2023) | 98 (77%completed) | 1 | 1 |
|  | Jarmo koli（2015） | 72 (90%completed) | 1 | 0 |
|  | Waller et al (2017) | 87 (87.36%completed) | 1 | 0 |
| Low-Intensity Training | Tom Arild Torstensen et al (2023) | 91 (77%completed) | 1 | 0 |
|  | Mohammed Alkatan (2016) | 48 (83.33%completed) | 1 | 1 |

**Supplementary Figure 2.** In comparison to land-based aerobic exercise, aquatic aerobic exercise demonstrates superior efficacy in pain management for patients, although land-based exercise can enhance knee joint function indices. A larger body of literature supports the notion that aquatic exercise is more effective in both alleviating pain and improving knee joint function.
