# Supplementary figures and images for: Clinical effect and mechanism of aerobic exercise for knee osteoarthritis: a mini review
Source: Front Physiol. 2025 Nov 12;16:1708750. doi: 10.3389/fphys.2025.1708750 (PMC12646927; doi:10.3389/fphys.2025.1708750)

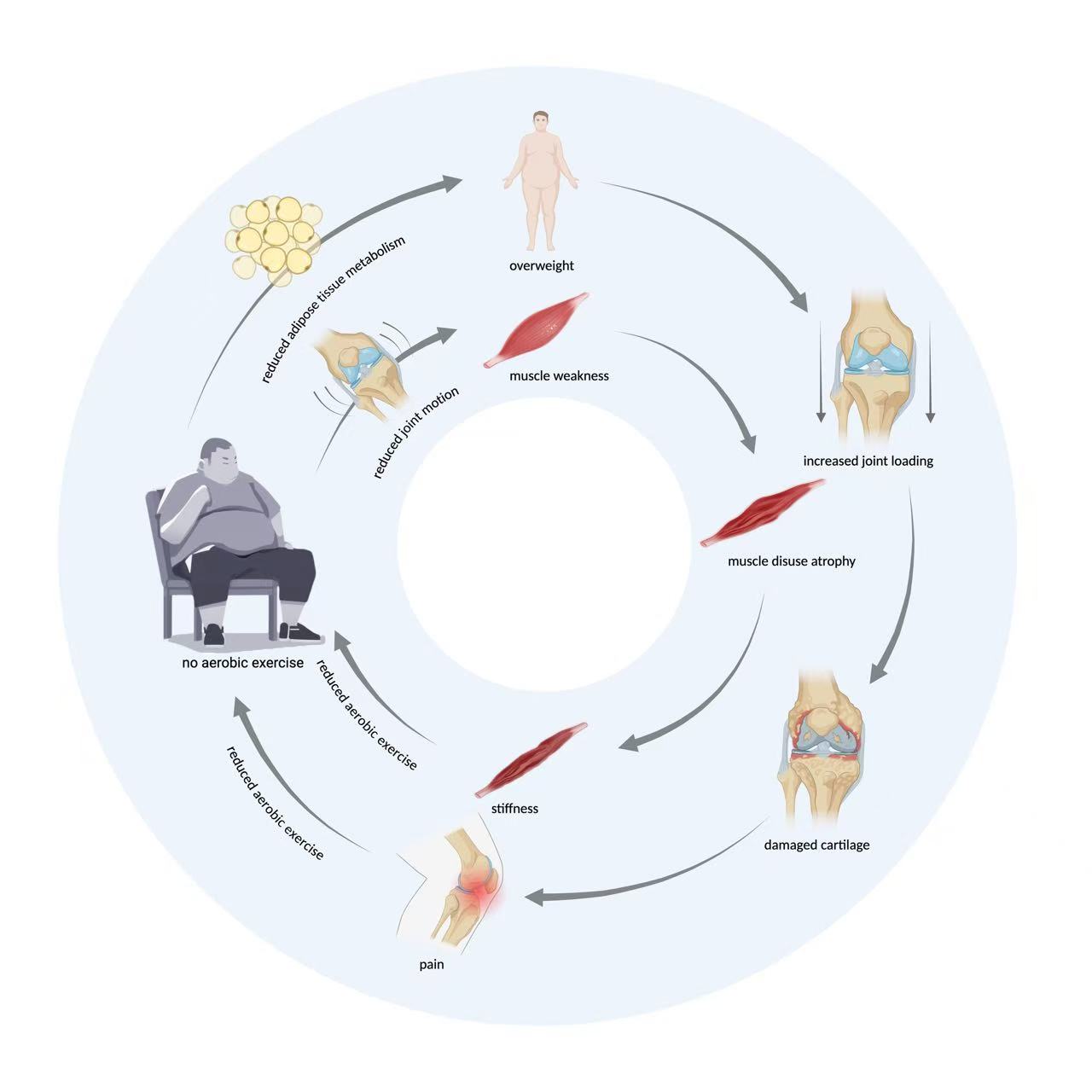

Supplement: Supplementary file 2 [file Image1.jpeg]
